# A Novel Cecropin-LL37 Hybrid Peptide Protects Mice Against EHEC Infection-Mediated Changes in Gut Microbiota, Intestinal Inflammation, and Impairment of Mucosal Barrier Functions

**DOI:** 10.3389/fimmu.2020.01361

**Published:** 2020-06-30

**Authors:** Xubiao Wei, Lulu Zhang, Rijun Zhang, Matthew Koci, Dayong Si, Baseer Ahmad, Junhao Cheng, Junyong Wang, Maierhaba Aihemaiti, Manyi Zhang

**Affiliations:** ^1^Laboratory of Feed Biotechnology, State Key Laboratory of Animal Nutrition, College of Animal Science and Technology, China Agricultural University, Beijing, China; ^2^Prestage Department of Poultry Science, College of Agriculture and Life Sciences, North Carolina State University, Raleigh, NC, United States

**Keywords:** enrofloxacin, *Escherichia coli*, hybrid peptide, inflammation, microflora, mucosal barrier, O157:H7

## Abstract

Intestinal inflammation can cause impaired epithelial barrier function and disrupt immune homeostasis, which increases the risks of developing many highly fatal diseases. Enterohemorrhagic *Escherichia coli* (EHEC) O157:H7 causes intestinal infections worldwide and is a major pathogen that induces intestinal inflammation. Various antibacterial peptides have been described as having the potential to suppress and treat pathogen-induced intestinal inflammation. Cecropin A (1–8)-LL37 (17–30) (C-L), a novel hybrid peptide designed in our laboratory that combines the active center of C with the core functional region of L, shows superior antibacterial properties and minimized cytotoxicity compared to its parental peptides. Herein, to examine whether C-L could inhibit pathogen-induced intestinal inflammation, we investigated the anti-inflammatory effects of C-L in EHEC O157:H7-infected mice. C-L treatment improved the microbiota composition and microbial community balance in mouse intestines. The hybrid peptide exhibited improved anti-inflammatory effects than did the antibiotic, enrofloxacin. Hybrid peptide treated infected mice demonstrated reduced clinical signs of inflammation, reduced weight loss, reduced expression of pro-inflammatory cytokines [tumor necrosis factor-alpha (TNF-α), interleukin-6 (IL-6), and interferon-gamma (IFN-γ)], reduced apoptosis, and reduced markers of jejunal epithelial barrier function. The peptide also affected the MyD88–nuclear factor κB signaling pathway, thereby modulating inflammatory responses upon EHEC stimulation. Collectively, these findings suggest that the novel hybrid peptide C-L could be developed into a new anti-inflammatory agent for use in animals or humans.

## Introduction

Commensal microflora in the intestinal mucosal can promote intestinal stability and prevent pathogens from invading the intestine. However, when the balance of intestinal microbiota is disturbed, the intestinal defense function and immunoregulatory functions dramatically decrease, which can cause intestinal inflammation (1–5). Intestinal inflammation is a defensive response to stimulation of the host by microbiological toxins or pathogens (1–4). Although a controlled inflammatory response is beneficial, it can become detrimental if the response fails to eliminate the pathogen or the inflammatory process persists for an extended period of time (6).

Within the intestinal epithelium, the mucosal barrier plays an important role in maintaining physiological homeostasis by physically blocking the passage of harmful foreign antigens, microbes, and their toxins into the host (5, 7). When this barrier function breaks down the intestinal contents and bacteria can diffuse past the mucosal layer, which can initiate host responses that lead to further break down of the epithelia barrier resulting in activation of the intestinal inflammatory response (5, 7). While not all bacteria induce inflammatory diseases, intestinal pathogens like enterohemorrhagic *E. coli* (EHEC) O157:H7 induce a particularly acute and potentially fatal infection if left untreated (8, 9). Even with treatment EHEC O157:H7 infection can lead to hemolytic-uremic syndrome and permanent kidney damage, especially in young children (8, 9).

Short-term, appropriate application of antibiotics is an option to reduce the intestinal bacterial load and intestinal inflammatory disease severity caused by overgrowth of pathogenic bacteria (10–14). Historically, the broad-spectrum antibiotic enrofloxacin (Enro) has been used to treat diseases during previous decades (10–13). However, antibiotic resistance has become a serious global issue and is steadily increasing worldwide in almost every species treated with antibiotics (15, 16). Also of increasing concern, long-term antibiotic treatment can alter the microecosystem balance by causing compositional changes in the intestinal microbiota and may lead to a homeostatic imbalance by altering the expression of tight junction proteins, mucin, antimicrobial peptides, and cytokines in intestinal epithelial cells (17, 18). Thus, it is necessary to explore novel safe antibiotic alternatives for inflammation therapy.

Recent findings suggest that specific antibacterial peptides (AMPs) elicit certain anti-inflammatory effects (19–24); specifically, Cecropin A (C) (19) and LL37 (L) (20). Previously, we generated a hybrid peptide, Cecropin A (1–8)-LL37 (17–30) (C-L), by combining the N-terminal cationic hydrophobic fragment of C with the core functional region of L and examined its properties (25). As compared to the parental peptides (C and L), the hybrid C-L peptide exhibited excellent antibacterial properties and was more effective against gram-positive and gram-negative bacteria (25). These results demonstrate that the hybrid C-L peptide may serve as a potential antibacterial pharmaceutical agent. Hence an important question arises as to whether the C-L peptide can prevent or attenuate intestinal inflammation induced by harmful gut bacteria while killing them.

In this study, we investigated whether the C-L peptide could provide effective therapy against intestinal inflammation and impairment of epithelial barrier function induced by EHEC O157:H7 and explore the underlying mechanisms, using a mouse model of intestinal inflammation.

## Materials and Methods

### Peptide Synthesis

The C-L peptide (KWKLFKKIFKRIVQRIKDFLRN) was chemically synthesized (95% purity) by KangLong Biochemistry (Jiangsu, China). The molecular weight of the C-L peptide was confirmed using a Thermo Finnigan LCQ ion-trap mass spectrometer (Thermo Finnigan, CA, USA). The peptide was then suspended in endotoxin-free water and stored at −80°C.

### Animal Model

Seventy-two C57/BL6 female mice (4 weeks of age) were purchased from Charles River Laboratories (Beijing, China) and maintained under standard conditions. The animal experiments were conducted with the approval of the China Agricultural University Animal Care and Use Committee (Beijing, China).

All animals had free access to feed and fresh water during the experimental period. EHEC O157:H7 (ATCC43889) strain was purchased from China Veterinary Culture Collection Center (Beijing, China) and cultured in Luria–Bertani (LB) broth. Mice were randomly divided into the following six groups (*n* = 12): the control, EHEC, EHEC+Enro, EHEC+C-LL (EHEC+low dose of C-L), EHEC+C-LM (EHEC+moderate dose of C-L), and EHEC+C-LH (EHEC+high dose of C-L) groups. The control group was orally administered 100 μL sterile phosphate-buffered saline (PBS); the EHEC group was orally administered 100 μL sterile PBS containing 1 × 10^8^ colony-forming units (CFUs) EHEC O157:H7; the EHEC+Enro group was orally administered 100 μL sterile PBS containing 1 × 10^8^ CFUs EHEC O157:H7 and then treated by intraperitoneal (i.p.) injection with 8 mg/kg Enro once/day for 3 days; the EHEC+C-LL, EHEC+C-LM, and EHEC+C-LH groups were administered 100 μL sterile PBS containing 1 × 10^8^ CFUs EHEC O157:H7 and then treated by i.p. injection with 4, 8, and 16 mg/kg C-L, respectively, once/day for 3 days. After 3 days, the mice were euthanized, and tissues were collected for analysis. The method used in this section is similar to that previously presented by Zhang et al. (26) and Wang et al. (27). Before and after the study, the body weights of mice were confirmed and recorded.

### Microbial Composition Analysis

Total bacterial DNA was extracted using cecal samples collected from the control, EHEC, EHEC+Enro, and EHEC+C-LM groups with the EZNA Stool DNA Kit (Omega Bio-tek, Norcross, GA, United States) according to the manufacturer's protocols. The DNA purity and yield were quantified using a NanoDrop 8000 spectrophotometer (Thermo Fisher Scientific, Scoresby, Australia). The V4 region of the bacterial 16S rRNA gene was amplified from the genome of cecal samples using the 515F (5′-GTGCCAGCMGCCGCGGTAA-3′) and 806R (5′-XXXXXXGGACTACHVGGGTWTCTAAT-3′) primer pair. All samples were sequenced on the Illumina MiSeq platform, according to standard protocols. Paired-end reads from the original DNA fragments were analyzed using fast-length adjustment of short reads (FLASH) software, version 1.2.8 (http://ccb.jhu.edu/software/FLASH). Operational taxonomic units (OTUs) were clustered using UPARSE (version 7.1, https://drive5.com/uparse/) at a 97% similarity level. Ribosomal Database Project (RDP) classifiers were applied to distribute 16S rRNA gene sequences into distinct taxonomic categories by aligning representative sequences with taxonomically annotated sequences.

### Induction of Intestinal Inflammation and Assessment of the Disease Activity Index (DAI)

EHEC-induced intestinal inflammation was assessed by determining the DAI, as described previously (23, 28). The DAI was determined as the sum of scores assigned for the body weight, stool consistency, and occult blood in the stool. Regarding body weights, the following scores were assigned: 0 for mice showing no weight loss, 1 for mice with 1–5% weight loss, 2 for mice with for 5–10% weight loss, 3 for mice with 10–20% weight loss, and four for mice with more than 20% weight loss. Stool consistency was scored as follows: 0, well-formed pellets; 2, pasty and semi-formed stools that did not adhere to the anus; and 4, liquid stools that adhered to the anus. For occult blood, a score of 0 was assigned for no blood, 2 was assigned for occult blood, and 4 was assigned for gross bleeding. For each mouse, the sum of these scores was divided by three, resulting in DAIs ranging from 0 (healthy) to 4 (maximal intestinal inflammation).

### Histopathology and Immunohistochemistry

Full-thickness sections of the middle jejunum were excised, dissected longitudinally, fixed immediately in 4% paraformaldehyde solution, and embedded in paraffin (26, 27). Samples were cut into 5-μm-thick sections, mounted on slides, and stained with hematoxylin and eosin (H&E). The epithelial morphological characteristics were observed microscopically (RM2235, Leica, Wetzlar, Germany). Chiu's scores were determined under blinded conditions using a histologic injury scale, as previously described (29, 30).

To prevent nonspecific binding during immunohistochemical analysis of CD177 expression, samples were blocked with PBS for 1 h containing 1% w/v bovine serum albumin (BSA) and incubated overnight at 4°C with anti-CD177 antibodies (Santa Cruz Biotechnology, CA, USA) at a dilution of 1:100. After washing three times with PBS, the samples were treated with horseradish peroxidase (HRP)-conjugated rabbit anti-goat IgG (Santa Cruz Biotechnology) at a ratio of 1:100. The samples were incubated at 4°C for 1 h and washed three times with PBS. Subsequently, the sections were treated with 3,3′-diaminobenzidine (DAB) substrate (50–100 μL; DAKO, Carpinteria, CA, USA) and stained with Harris hematoxylin. Finally, the samples were dehydrated with an alcohol gradient (70–100%), and xylene was used to increase the transparency of the slides. A neutral balsam was applied for mounting.

The apoptotic cells in the jejunal sections were detected via the terminal deoxynucleotidyl transferase dUTP nick-end labeling (TUNEL) method using a TUNEL staining kit (Roche, Indianapolis, IN, USA) according to the manufacturer's instructions. The sections were co-stained with the 4′,6-diamidino-2-phenylindole (DAPI; Servicebio, Wuhan, China), and the number of apoptotic cells was counted in 4–6 randomly selected fields at 200× magnification.

### Cytokine and Myeloperoxidase (MPO)-Activity Assay

Interleukin-6 (IL-6), interferon-gamma (IFN-γ), and tumor necrosis factor (TNF-α) levels were qualified in the jejunum, using commercial enzyme-linked immunosorbent assay (ELISA) kits (eBioscience, San Diego, CA, USA). The MPO activities in the jejunum were determined using an ELISA kit (Boster, Wuhan, China). Samples were assessed according to the manufacturers' instructions.

### Western Blotting

Jejunum tissues were ground and lysed using a Total Protein Extract Kit (KeyGEN Biotech, Nanjing, China) according to manufacturer's instructions. Proteins in each supernatant were separated by 10% sodium dodecyl sulfate-polyacrylamide gel electrophoresis and transferred onto a nitrocellulose membrane. Next, the membranes were blocked with 5% (w/v) skim dried milk proteins in 0.05% (w/v) TBST and immunoblotted overnight with primary antibodies against nuclear factor-κ-gene binding (NF-κB; p65), phospho-NF-κB (p-NF-κB; p-p65), inhibitory subunit of NF-κB (IκB)-α, phosphor-IκB-α (p-IκB-α), inhibitor of NF-κB kinase (IKK)-β, phosphor-IKK-β (p-IKK-β), ZO-1, occluding, or β-actin (Santa Cruz, CA, USA) at 4°C. After washing with TBST, the proteins of interest were labeled with HRP-conjugated secondary antibodies (HuaAn, Hangzhou, China) for 1 h. Bands were detected using the SuperSignal West Femto maximum sensitivity substrate (Pierce Biotechnology, Rockford, Illinois, USA).

### Electrophysiology Measurements

The transepithelial-electrical resistance (TEER) values of intestinal membranes were evaluated using an *in vitro*-diffusion chamber method and stripped mice jejunal membranes. After removing the underlying muscularis of the intestinal membranes, the intestinal segments were mounted in a diffusion chamber (World Precision Instruments; Narco Scientific, Mississauga, Ontario, Canada) equipped with two pairs of Ag/AgCl electrodes connected to the chambers via 3 M KCl/3.5% agar bridges. This method was used to identify and quantify the potential difference (PD), short-circuit current (Isc), and total electrical resistance (RT). Electrical resistance was calculated according to Ohm's law (Equation 1) (31):

(1) $$\begin{array}{l} RT=\text{PD}/\text{Isc} \end{array}$$

### Immunofluorescence Analysis of Tight Junction (TJ) Proteins

The expression level of the intercellular TJ protein zonula occludens-1 (ZO-1) was detected. Nonspecific binding sites were blocked with PBS containing 1% w/v BSA for 30 min at 25°C. The samples were incubated with an anti-ZO-1 antibody (Santa Cruz Biotechnology) overnight at 4°C. The slices were washed several times with PBS, followed by incubation with a tetramethylrhodamine isothiocyanate-conjugated secondary antibody (Santa Cruz Biotechnology) at a ratio of 1:100 for 1 h at 25°C in the dark. Nuclei were stained with DAPI. For the mounting of the samples onto slides, Glycerol was used. Images were acquired with a fluorescence microscope (BZ-800; Keyence, Osaka, Japan).

### Transmission Electron Microscopy (TEM)

Epithelial cell junctions and microvilli were characterized by TEM. For TEM processing, jejunum tissues were fixed with 2.5% glutaraldehyde. After washing, the samples were treated with 2% osmium tetroxide buffer and then placed in 0.5% aqueous uranyl acetate. The tissue sections were washed with 50% alcohol and then dehydrated using a graded ethanol series. Samples were embedded in eponate. Serial ultrathin sections were cut and imaged on a TEM (H-7650, Hitachi, Japan).

### Bacterial Transfer During EHEC Infection

After the mice were treated as described in section Animal Model above, 10 mice each from the control, EHEC, EHEC+Enro, and EHEC+C-LM groups were randomly selected. The mice were euthanized, and their livers and spleens were collected and homogenized in cold PBS. The amount of CFUs were quantified by plating serial dilutions on Luria–Bertani agar plates.

### Statistics

The data are presented as mean ± standard deviation from a minimum of three independent experiments. Statistical comparisons were carried out using Student's *t-*tests and one-way analysis of variance using GraphPad Prism v6 software (La Jolla, California). A *P* ≤ 0.05 was considered as significant. NS: *P* > 0.05, ^*^P ≤ 0.05, ^**^P ≤ 0.01, ^***^P ≤ 0.001, ^****^P ≤ 0.0001.

## Results

### C-L Treatment Improved the Microbiota Composition and Microbial Community Balance in Mouse Intestines

**Figure 1:**
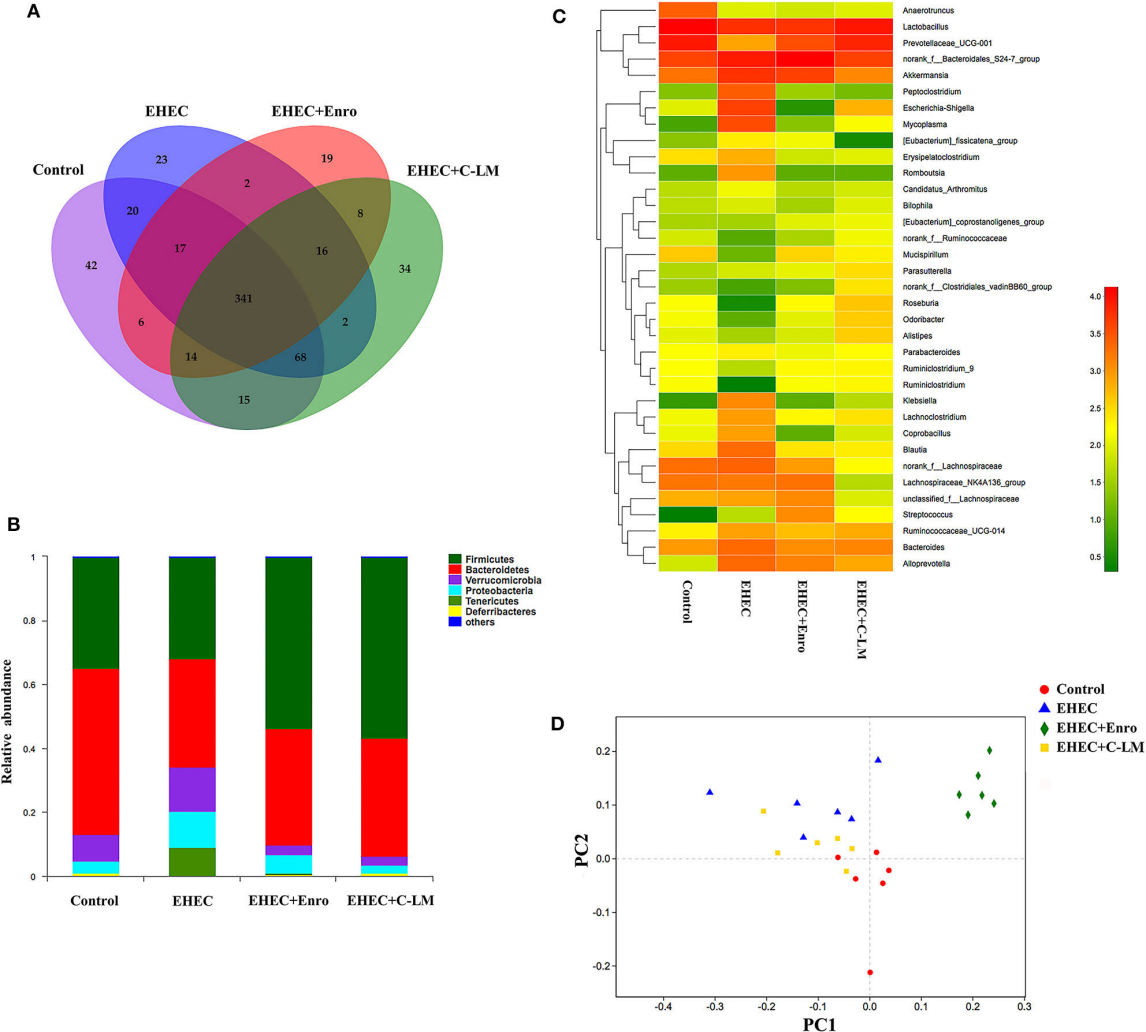
C-L improved the microbiota composition and microbial community balance in mouse intestines. The cecal microbial communities in mice were investigated by 16S rRNA gene sequencing, using the Illumina platform (*n* = 6). **(A)** Venn diagram showing the unique and common OTUs in the mouse intestine. **(B)** Relative abundance of the most abundant bacterial phyla. **(C)** Heat map constructed with the top 35 genera. **(D)** Nonmetric multidimensional scaling based on the OTU levels. The control group was orally administered 100 μL sterile PBS; the EHEC group was orally administered 100 μL sterile PBS containing 1 × 10^8^ CFUs EHEC O157:H7; the EHEC+Enro and EHEC+C-LM groups were administered 100 μL sterile PBS containing 1 × 10^8^ CFUs EHEC O157:H7 and then treated by i.p. injection with 8 mg/kg Enro and C-L, respectively, once/day for 3 days.

We examined the effects of EHEC infection, with and without C-L and Enro treatment on the microbiota composition in mouse ceca by Illumina sequencing of the 16S rRNA V4 region. A Venn diagram was constructed to show the numbers of common and unique OTUs (Figure 1A) (32). The unique OTUs in the control, EHEC, EHEC+Enro, and EHEC+C-LM groups were 42, 23, 19, and 34, respectively; the total number of OTUs in each sample was 523, 489, 423, and 498, respectively. Thus, the ratios of unique to total OTUs were 8, 4.7, 4.4, and 6.8%, respectively (Figure 1A). Firmicutes, Bacteroidetes, Verrucomicrobia, and Proteobacteria were the four most abundance bacterial phyla among all groups, and both C-L and Enro reduced EHEC-dependent Proteobacteria induction and overcame EHEC-induced Firmicutes and Bacteroidetes downregulation (Figure 1B). At the genus level, 35 genera were identified (Figure 1C). *Lactobacillus, Prevotellaceae*, and *Akkermansia* species were the most prominent in the control, EHEC+Enro, and EHEC+C-LM groups. EHEC infection lead to a significant increase of *Peptoclostridium, Escherichia-Shigella, Klebsiella, Lachnoclostridium, Coprobacillus, Blautia*, and *Lachnospiraceae* species, C-L and Enro administration significantly downregulated these species, and C-L showed stronger downregulation than Enro (Figure 1C). Additionally, using nonmetric multidimensional scaling analysis, we found that samples of the EHEC+Enro group formed a unique cluster and separated from the other groups (Figure 1D), suggesting that Enro may adversely affect the microbial composition.

### Effect of C-L on the Body Weight and DAI

**Figure 2:**
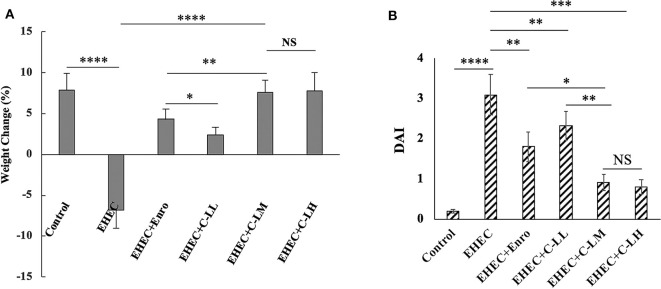
Effect of C-L on the body weight **(A)** and DAI **(B)**. The EHEC+C-LL, EHEC+C-LM, EHEC+C-LH, and EHEC+Enro groups were orally administered 100 μL sterile PBS containing 1 × 10^8^ CFUs EHEC O157:H7 and then treated by i.p. injection with 4, 8 mg/kg, or 16 mg C-L/kg body weight C-L, or 8 mg Enro/kg body weight, respectively, once/day for 3 days. The control and EHEC groups were orally administered 100 μL sterile PBS or 100 μL sterile PBS containing 1 × 10^8^ CFUs EHEC O157:H7, respectively, without any follow-up treatments. The DAI value was derived from scores relating to weight loss, stool consistency, and occult blood. The data are shown as the mean ± standard deviation (*n* = 12). NS, *P* > 0.05; *P ≤ 0.05; **P ≤ 0.01; ***P ≤ 0.001; ****P ≤ 0.0001.

Based on the body weight and DAI data, we concluded that the mouse intestinal inflammation model was successfully established (Figure 2). As expected, Mice in the EHEC-treated group had significantly more weight loss than did those in the control group, whereas mice in other groups had less weight loss compared with mice in the EHEC-treated group (Figure 2A). Notably, mice in the C-L plus EHEC groups demonstrated less weight loss as compared to the EHEC group, in the case of C-LM and C-LH less weight loss than the Enro control. In addition to body weight loss, EHEC infection induced several other gross clinical changes. All of these clinical signs (weight loss, blood in the perianal region, and the presence of diarrhea) were scored and the scores combined into a DAI to holistically assess the impact C-L treatment had on EHEC induced disease. In contrast with the EHEC-treated group, which showed a significantly higher DAI value than the control group, mice in the EHEC+C-LM group apparently recovered, as reflected by the reduced DAI value. Furthermore, the DAI value of the EHEC+C-LM group was markedly lower than that of the EHEC+Enro group (Figure 2B).

### The Protective Effects of C-L Against EHEC-Induced Damage in Intestinal Tissue

**Figure 3:**
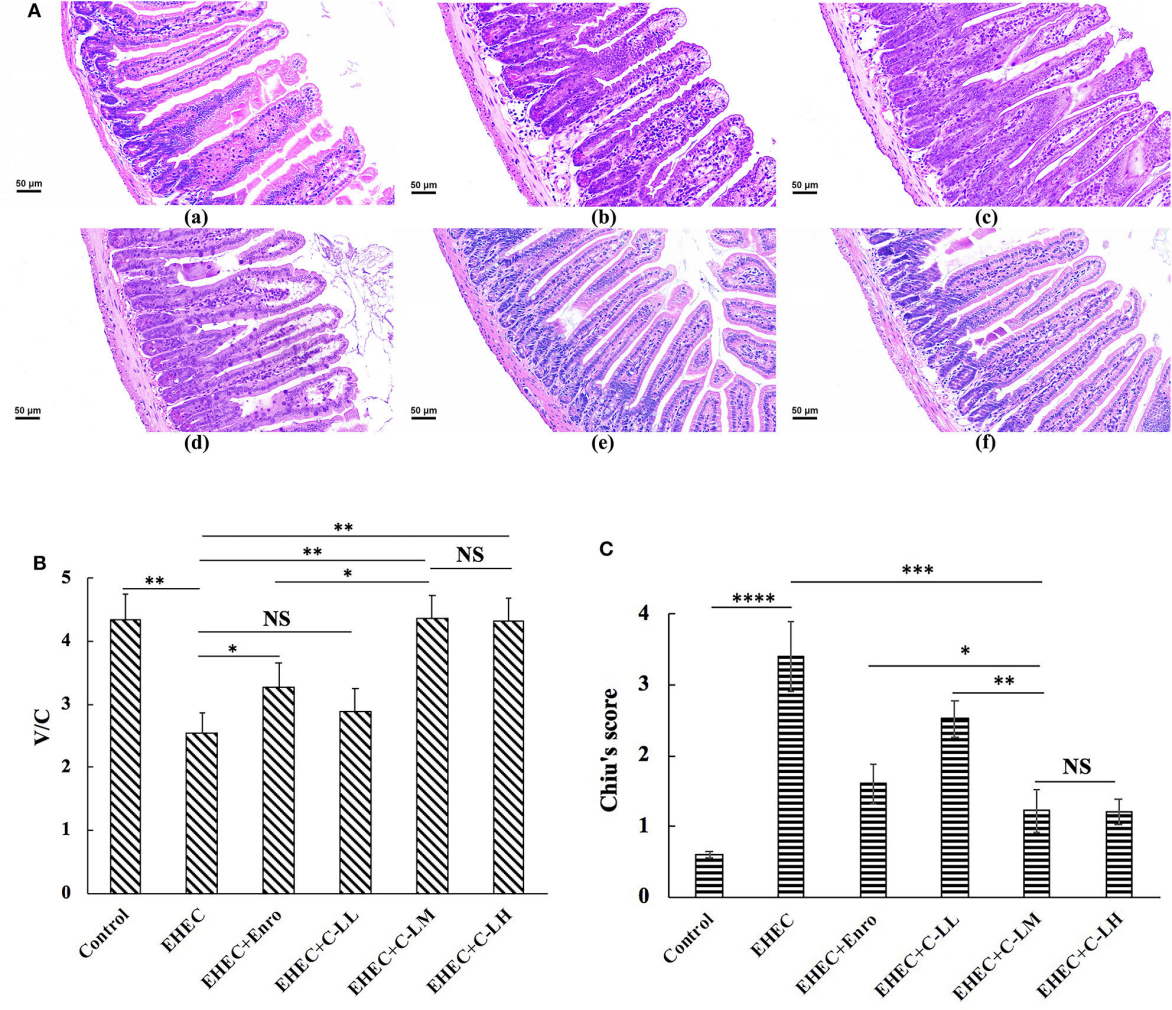
The protective effects of C-L against EHEC-induced clinical symptoms in the mouse jejunum. Representative H&E-stained sections from mice in the (**A**-a) control, (**A**-b) EHEC, (**A**-c) EHEC+Enro, (**A**-d) EHEC+C-LL, (**A**-e) EHEC+C-LM, and (**A**-f) EHEC+C-LH groups. Scale bar, 50 μm. **(B)** The effect of C-L on the jejunum V/C ratio. **(C)** The effect of C-L on Chiu's score. The control group was orally administered 100 μL sterile PBS; the EHEC group was orally administered 100 μL sterile PBS containing 1 × 10^8^ CFUs EHEC O157:H7; the EHEC+Enro group was orally administered 100 μL sterile PBS containing 1 × 10^8^ CFUs EHEC O157:H7 and then treated by i.p. injection with 8 mg/kg Enro once/day for 3 days; the EHEC+C-LL, EHEC+C-LM, and EHEC+C-LH groups were administered 100 μL sterile PBS containing 1 × 10^8^ CFUs EHEC O157:H7 and then treated by i.p. injection with 4, 8, and 16 mg/kg C-L, respectively, once/day for 3 days. The data are shown as the mean ± standard deviation (*n* = 8). NS, *P* > 0.05; *P ≤ 0.05; **P ≤ 0.01; ***P ≤ 0.001; ****P ≤ 0.0001.

EHEC infection lead to damage to the jejunal epithelium and intestinal inflammation (Figure 3). H&E staining of jejunum tissue revealed EHEC infection caused considerable villi blunting, thicker serosa, infiltration of lymphocytes, and edema (Figure 3A). In contrast, tissues sections from mice treated with peptide or antibiotic were observed to have reduced signs of damage to the epithelium (Figure 3A).

As shown in Figure 3B, the villus height/crypt depth ratio (V/C) in the EHEC-infected group decreased markedly as compared to the control group. In contrast, mice in the EHEC+Enro, EHEC+C-LM, and EHEC+C-LH groups had significantly higher V/C values than mice in the EHEC group. Moreover, the C-L peptide more potently improved the V/C ratio than did Enro at the same concentration. Additionally, no differences were detected between the EHEC-infected group and the EHEC+C-LL group (Figure 3B). Chiu's score in the C-LM-treated group was also markedly lower than that in the antibiotic-treated group. Overall, our data indicated that C-L treatment significantly attenuated EHEC-induced intestinal damage (Figure 3C).

**Figure 4:**
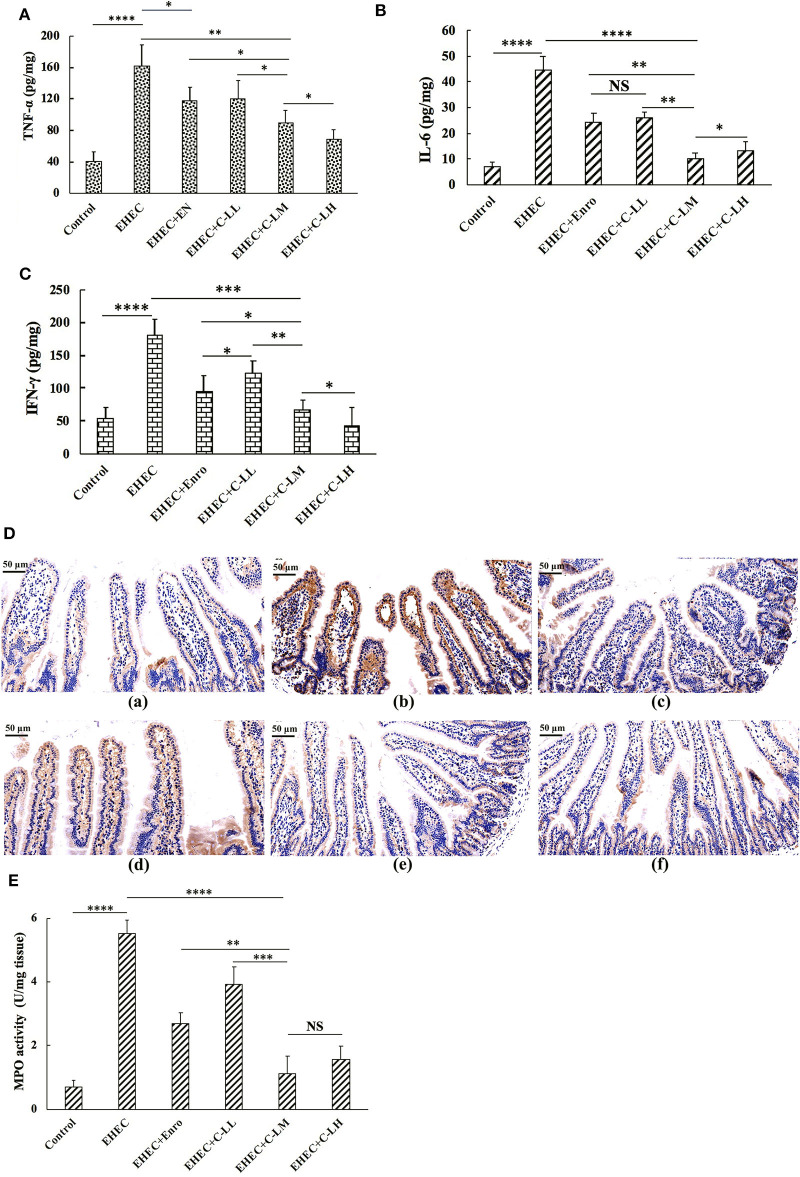
The protective effects of C-L on inflammatory response. The expression levels of TNF-α **(A)**, IL-6 **(B)**, and IFN-γ **(C)** in mouse jejunum tissues were measured by ELISA. **(D)** Representative images of CD177^+^ cells in the (**D**-a) control, (**D**-b) EHEC, (**D**-c) EHEC+Enro, (**D**-d) EHEC+C-LL, (**D**-e) EHEC+C-LM, and (**D**-f) EHEC+C-LH groups. Scale bar, 50 μm. Enzymatic activities of MPO were measured **(E)**. The control group was orally administered 100 μL sterile PBS; the EHEC group was orally administered 100 μL sterile PBS containing 1 × 10^8^ CFUs EHEC O157:H7; the EHEC+Enro group was orally administered 100 μL sterile PBS containing 1 × 10^8^ CFUs EHEC O157:H7 and then treated by i.p. injection with 8 mg/kg Enro once/day for 3 days; the EHEC+C-LL, EHEC+C-LM, and EHEC+C-LH groups were administered 100 μL sterile PBS containing 1 × 10^8^ CFUs EHEC O157:H7 and then treated by i.p. injection with 4, 8, and 16 mg/kg C-L, respectively, once/day for 3 days. The data are shown as the mean ± standard deviation (*n* = 8). NS, *P* > 0.05; *P ≤ 0.05; **P ≤ 0.01; ***P ≤ 0.001; ****P ≤ 0.0001.

To evaluate the inhibitory effects of C-L and Enro on the EHEC-induced inflammatory response, the expression levels of the pro-inflammatory cytokines TNF-α (Figure 4A), IL-6 (Figure 4B), and IFN-γ (Figure 4C) in mice jejunum tissues were measured by ELISA. As shown in Figures 4A–C, the TNF-α, IL-6, and IFN-γ levels in the EHEC+C-LM and EHEC+Enro groups were significantly lower than those in the EHEC-infected group. In addition, mice that were infected with EHEC in the presence of 8 mg/kg C-L (C-LM) showed markedly lower TNF-α, IL-6, and IFN-γ expression than Enro-treated mice.

Immunohistochemical analysis demonstrated that mice treated with C-L (8 or 16 mg/kg) or Enro, the EHEC-infected group had significantly lower neutrophil infiltration than the group infected with EHEC alone (Figure 4D). These qualitative observations were then verified using a quantitative MPO ELISA to assay for MPO levels in the jejunum. These results demonstrate a clear increased in MPO in EHEC-infected mice as compared with control mice, with reduced levels of MPO in conjunction with C-L or Enro treatment, most notably in the EHEC+C-LM and EHEC+C-LH groups (Figure 4E).

**Figure 5:**
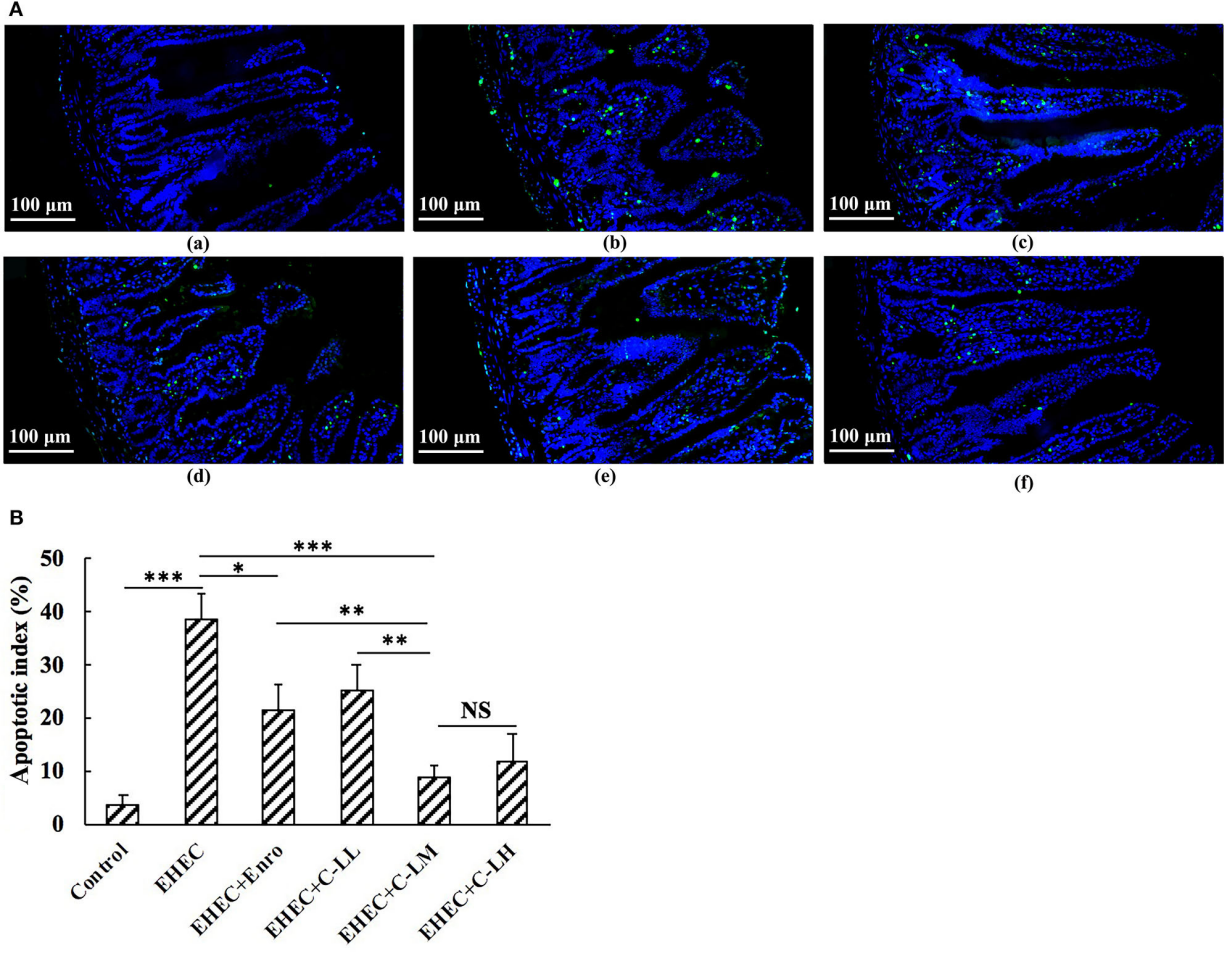
TUNEL staining of jejunum tissues from the panel (**A**-a) control, (**A**-b) EHEC, (**A**-c) EHEC+Enro, (**A**-d) EHEC+C-LL, (**A**-e) EHEC+C-LM, and (**A**-f) EHEC+C-LH groups. **(B)** The numbers of apoptotic cells were counted based on the average number of positive (green) cells. Bar, 100 μm. The control group was orally administered 100 μL sterile PBS; the EHEC group was orally administered 100 μL sterile PBS containing 1 × 10^8^ CFUs EHEC O157:H7; the EHEC+Enro group was orally administered 100 μL sterile PBS containing 1 × 10^8^ CFUs EHEC O157:H7 and then treated by i.p. injection with 8 mg/kg Enro once/day for 3 days; the EHEC+C-LL, EHEC+C-LM, and EHEC+C-LH groups were administered 100 μL sterile PBS containing 1 × 10^8^ CFUs EHEC O157:H7 and then treated by i.p. injection with 4, 8, and 16 mg/kg C-L, respectively, once/day for 3 days. The data are shown as the mean ± standard deviation (*n* = 8). NS, *P* > 0.05; *P ≤ 0.05; **P ≤ 0.01; ***P ≤ 0.001.

Jejunum tissue sections were analyzed by TUNEL staining to assay for changes in EHEC induced apoptosis (Figure 5). These results demonstrate a significant increase in apoptosis (green signals) in the EHEC-infected group as compared to the control group (Figure 5). Compared with the EHEC-infected group, C-L or Enro treated mice had significantly lower apoptotic indices (Figure 5B). Notably, the apoptosis index in mice treated with C-LM was similar to that in mice treated with C-LH, which was significantly lower than those for the mice in the Enro- and C-LL-treated groups (Figure 5B).

### The Effects of C-L on The MyD88–NF-κB Signaling Pathway in EHEC-Infected Mice

**Figure 6:**
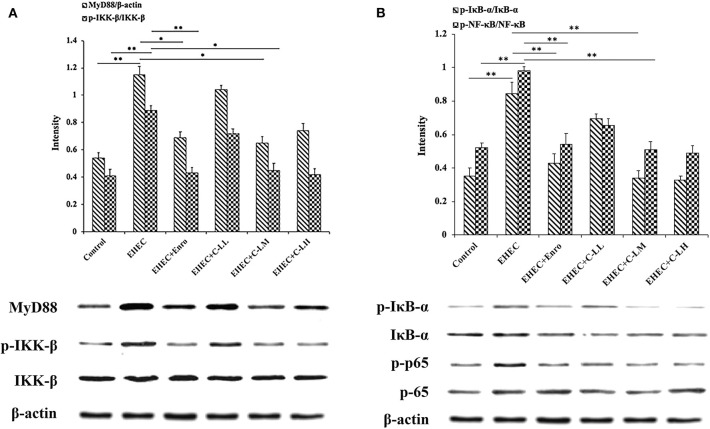
C-L inhibited the NF-κB (p65)-signaling pathway in mouse jejunum tissues. The same control image is used in the left **(A)** and right **(B)** columns. The control group was orally administered 100 μL sterile PBS; the EHEC group was orally administered 100 μL sterile PBS containing 1 × 10^8^ CFUs EHEC O157:H7; the EHEC+Enro group was orally administered 100 μL sterile PBS containing 1 × 10^8^ CFUs EHEC O157:H7 and then treated by i.p. injection with 8 mg/kg Enro once/day for 3 days; the EHEC+C-LL, EHEC+C-LM, and EHEC+C-LH groups were administered 100 μL sterile PBS containing 1 × 10^8^ CFUs EHEC O157:H7 and then treated by i.p. injection with 4, 8, and 16 mg/kg C-L, respectively, once/day for 3 days. Data are given as the mean ± standard deviation (*n* = 5). *P ≤ 0.05; **P ≤ 0.01.

To investigate the mechanism of C-L in modulating intestinal inflammation in mice stimulated with EHEC, the MyD88–NF-kB-signaling was examined (Figure 6). MyD88 levels and IKK-β, NF-κB (p65), and IκB-α phosphorylation in the jejunum increased significantly after infection with EHEC (Figures 6A,B). In contrast, the results showed that MyD88 expression and IKK-β, NF-κB, and IκB-α phosphorylation were suppressed in the jejunum following C-L treatment.

### C-L Prevented EHEC-Induced Disruption of The Intestinal TJ Structure and Function

**Figure 7:**
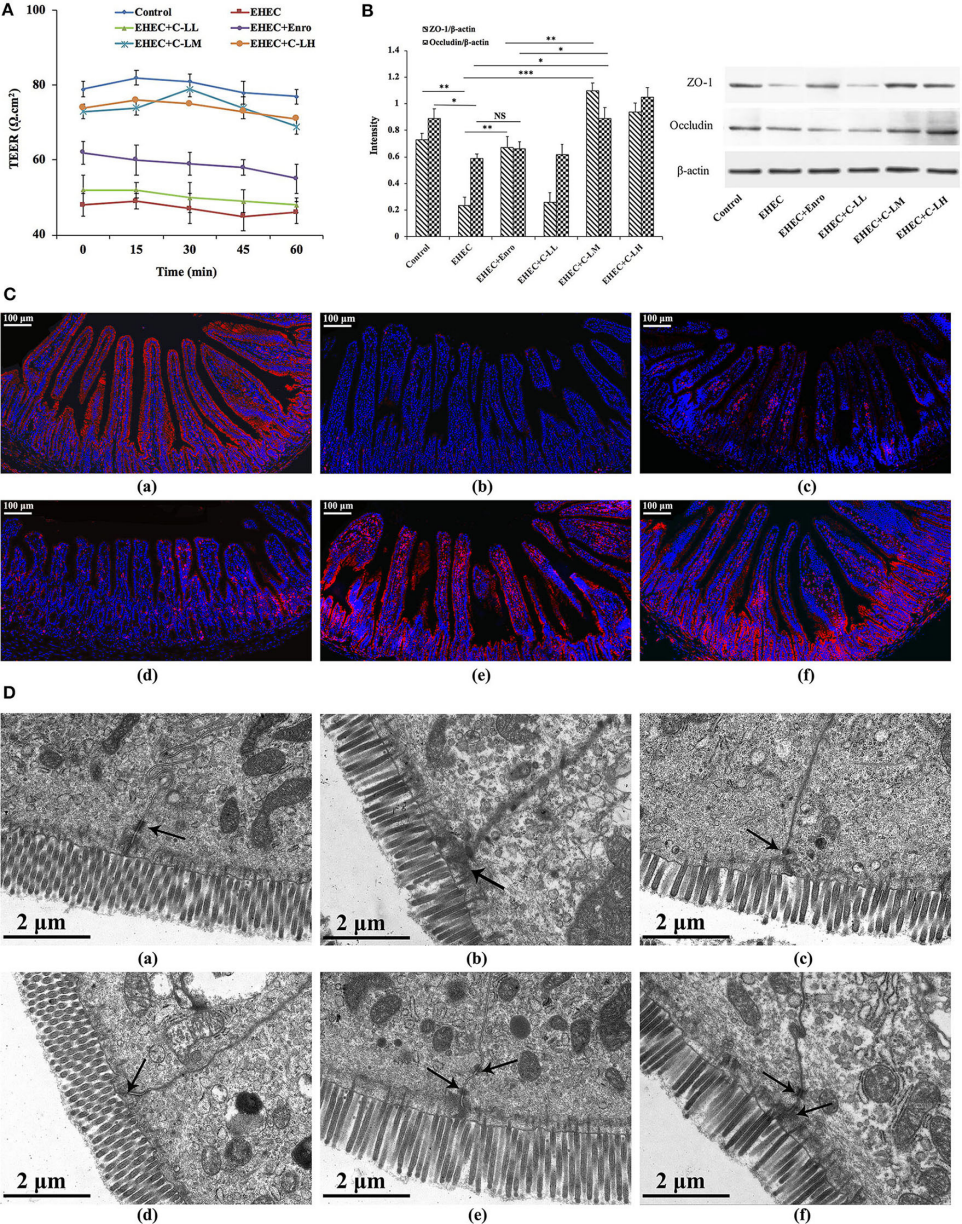
The protective effects of C-L on the intestinal barrier. **(A)** TEER values in mouse jejunum epithelium were detected using a Ussing chamber. **(B)** Expression levels of epithelial barrier function-related proteins (occluding and ZO-1) were determined by western blotting. **(C)** For immunofluorescence staining of occludin (red color) in jejunum tissue, stained slides with the (**C**-a) Control, (**C**-b) EHEC, (**C**-c) EHEC+Enro, (**C**-d) EHEC+C-LL, (**C**-e) EHEC+C-LM, (**C**-f) EHEC+C-LH groups were observed under a fluorescence microscope. Scale bar, 100 μm. **(D)** The protective effects of C-L on intestinal tight junction structures were examined by TEM for the (**D**-a) Control, (**D**-b) EHEC, (**D**-c) EHEC+Enro, (**D**-d) EHEC+C-LL, (**D**-e) EHEC+C-LM, (**D**-f) EHEC+C-LH groups. Narrower intervals and clearer desmosomes (black arrows) between the intestinal epithelial cells were found in C-L-treated mice. Scale bar, 2 μm. The control group was orally administered 100 μL sterile PBS; the EHEC group was orally administered 100 μL sterile PBS containing 1 × 10^8^ CFUs EHEC O157:H7; the EHEC+Enro group was orally administered 100 μL sterile PBS containing 1 × 10^8^ CFUs EHEC O157:H7 and then treated by i.p. injection with 8 mg/kg Enro once/day for 3 days; the EHEC+C-LL, EHEC+C-LM, and EHEC+C-LH groups were administered 100 μL sterile PBS containing 1 × 10^8^ CFUs EHEC O157:H7 and then treated by i.p. injection with 4, 8, and 16 mg/kg C-L, respectively, once/day for 3 days. The data are shown as the mean ± standard deviation (*n* = 5). NS, *P* > 0.05; *P ≤ 0.05; **P ≤ 0.01; ***P ≤ 0.001.

To characterize the effects of C-L on the functional integrity of the mouse intestinal epithelium, TEER values were evaluated over a 60 min period (Figure 7A). EHEC infection reduced the TEER values remarkably, indicating that permeability had increased. In contrast, tissues from C-LM or C-LH treated mice demonstrated TEER values ~80% of the control, confirming the role of C-L activation in minimizing EHEC-induced intestinal epithelial damage (Figure 7A). Tissues from Enro treated mice also demonstrated higher TEER values than the EHEC alone group, but its effect was significantly less than C-LM.

The expressions of specific TJ marker proteins (e.g., occluding and ZO-1) were analyzed by western blotting to further assess the impact of EHEC-induced damage to epithelial barrier function, and to what extent C-L treatment might ameliorate that damage (Figure 7B). TJ marker expression levels were downregulated in mice infected with EHEC alone, compared with control animals. Notably, treatment with C-LM and C-LH appeared to prevent the ZO-1 and occludin change in expression following EHEC-infection, whereas the expression levels of these TJ markers in the EHEC+Enro group was significantly lower than the EHEC+C-LM group (Figure 7B). These protective effects were further verified by immunofluorescent examination of the jejunum tissue (Figure 7C). EHEC infection significantly decreased occludin expression compared with the control group, whereas C-L treatment (8 mg/kg in particular) markedly increased occludin protein expression (Figure 7C).

Consistently, the tight junctions between the intestinal epithelial cells were verified by TEM, and these results confirmed that C-L protected against EHEC-induced damage in jejunum tissues (Figure 7D).

### C-L Decreased EHEC-Induced Bacterial Transfer

**Figure 8:**
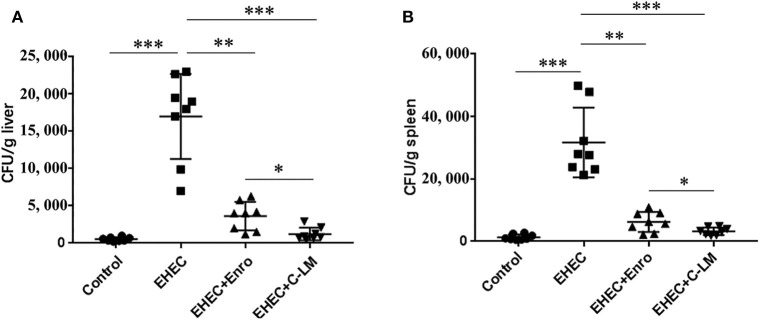
Improvement of host defense against EHEC infection by C-L. The number of bacterial CFUs transferred to the liver **(A)** and spleen **(B)** on day 3 after infection are shown. The control group was orally administered 100 μL sterile PBS; the EHEC group was orally administered 100 μL sterile PBS containing 1 × 10^8^ CFUs EHEC O157:H7; the EHEC+Enro and EHEC+C-LM groups were administered 100 μL sterile PBS containing 1 × 10^8^ CFUs EHEC O157:H7 and then treated by i.p. injection with 8 mg/kg Enro and C-L, respectively, once/day for 3 days. The data are shown as the mean ± standard deviation (*n* = 5). NS, *P* > 0.05; *P ≤ 0.05; **P ≤ 0.01; ***P ≤ 0.001.

EHEC infection significantly increased the transfer of bacteria to the spleen and liver compared to the control group, which was effectively attenuated by Enro or C-L treatment (Figure 8). In addition, C-L was more effective than Enro at the same concentration.

## Discussion

Bacterial infection is responsible for many serious and fatal diseases. Bacteria can elicit mucosal immune responses, and if unresolved can lead to a breakdown in mucosal homeostasis and inflammation (2). Microbial pathogens, such as EHEC, could induce severe intestinal inflammation leading to severe systemic complications (3, 8, 9). Antibiotics, such as Enro (10–13), have been commonly used to treat inflammation, based on their antibacterial activities (33). However, as the incidence of antibiotic-resistant increases worldwide (34, 35), the need to develop new classes of anti-inflammatory compounds to fight tissue inflammation also increases.

Previously, it was reported that specific AMPs elicit anti-inflammatory effects (19–23), such as C (19) and L (20). These AMPs merit consideration as clinical alternatives against the rising threat of inflammation. Hybridization is a novel method for designing new peptides to combine the advantages of different native peptides. The novel hybrid peptide C-L, designed in our laboratory, showed significantly increased antimicrobial activity and minimized cytotoxicity, compared to the parental peptides (C and L), suggesting that it has tremendous potential for use as a novel anti-inflammatory agent (25).

Murine models of intestinal inflammation have been extensively used to investigate the regulatory mechanisms that relieve inflammation and maintain intestinal homeostasis (36). In this study, we established an EHEC-induced mouse model to investigate the anti-inflammatory activity of C-L and its potential as a new therapeutic to replace or supplement antibiotics.

Intestinal microbes form a symbiotic ecosystem that helps maintain the homeostatic balance in the gut, and is indispensable for human and animal health (37). It was reported that alterations in the bacterial flora of the intestine can cause intestinal inflammation (38, 39). With Illumina sequencing of the 16S rRNA gene, we found that the microbial diversity differed significantly between EHEC-infected mice and non-infected controls. The microbiota of EHEC-infected mice displayed reduced levels of two phyla of bacteria, *Firmicutes* and *Bacteriodetes*, compared with non-infected controls. The ability to increase the levels of *Firmicutes* and *Bacteriodetes* in the gut might enable C-L and Enro to develop anti-inflammatory activity. This possibility was supported by the study of Scanlan et al. (40) who speculated that the decrease in *Firmicutes* and *Bacteriodetes* may be related to the occurrence of intestinal inflammation. A substantial body of evidence has demonstrated that *Peptoclostridium* (41, 42), *Escherichia-Shigella* (41), *Klebsiella* (43), *Lachnoclostridium* (44), *Blautia* (45, 46), and *Lachnospiraceae* (46, 47) species are important drivers of intestinal inflammation. In this study, treatment with C-L and Enro effectively inhibited the increase of *Peptoclostridium, Escherichia-Shigella, Klebsiella, Lachnoclostridium, Blautia*, and *Lachnospiraceae* species induced by EHEC infection, suggesting that C-L has the potential to inhibit EHEC-induced enteritis by inhibiting the growth of inflammatory pathogens in the intestine. In addition, the EHEC+Enro and control groups had significantly different OTU values, and compared to the EHEC+C-LM group, the OTU values of the group treated with C-L were better and closer to that of the control group than the group treated with Enro. Further, after Enro treatment, the bacterial intestinal flora formed unique clusters that separated from the other groups, suggesting that antibiotic treatment may have distinct negative effects on the microbial composition.

We further examined the anti-inflammatory activity of C-L. We found that both Enro and C-L treatments could result in efficient protection against EHEC-induced damage, as assessed by the DAI values, Chiu's scores, and histological damage to the intestines. The villus height:crypt depth ratios, which were closely associated with the hosts' growth performance and intestinal absorption, were significantly improved by C-L administration to EHEC-induced mice. Additionally, administering C-L or Enro ameliorated EHEC-induced intestinal inflammation and effectively decreased the infiltration of activated neutrophils, which can produce superoxide anions and other reactive species, leading to the formation of highly reactive hydroxyl radicals that may contribute significantly to tissue necrosis and mucosal dysfunction (48–50). The MPO activity level is directly proportional to the concentration of neutrophils in inflamed tissues and is, thus, an index of inflammation and neutrophil infiltration (51). Consistently, our findings showed that treatment with C-L or Enro significantly reduced MPO activity in jejunum tissues in EHEC-infected mice.

Intestinal inflammation is regulated by release of pro-inflammatory cytokines, such as TNF-α, IL-6, and IFN-γ (52, 53). In our study, the expression of these pro-inflammatory cytokines was significantly suppressed by Enro and C-L. Notably, C-L inhibited inflammatory cytokine production more potently than Enro at the same concentration.

Numerous studies have identified important functions for necroptosis in inflammation and have suggested that it could be implicated in the pathogenesis of many inflammatory diseases (54). It was reported apoptosis is one of the ulcerogenic processes associated with intestinal inflammation (53). In addition, apoptosis is active in hosts with inflammatory bowel disease (IBD) and heightens intestinal inflammation (54–56). In this study, TUNEL staining showed that EHEC stimulation robustly increased apoptosis in mouse intestinal cells. C-L dose-dependently inhibited apoptosis in EHEC-infected mice. Enro-treated mice showed less potent inhibition of EHEC-induced apoptosis at the same concentrations.

It was reported that the NF-κB signaling is a principal pathway that regulates cytokines, such as IL-1β, IL-6, and TNF-α, and cells that participate in inflammatory process (57). Activation of the MyD88-dependent pathway leads to IKK and NF-κB phosphorylation, eventually contributing to pro-inflammatory cytokine expression (58). Given this background, we decided to assess the phosphorylation levels of key factors involved in the NF-κB-signaling pathway. The results showed that IKK-β, IκB-α, and p65 phosphorylation was typically increased in the small intestines of mice after EHEC infection, whereas EHEC+C-L treatment effectively downregulated the phosphorylation level of these proteins. In addition, C-L treatment decreased MyD88 expression in the jejunum. Collectively, these findings indicated that C-L could prevent EHEC-induced intestinal inflammation by inhibiting the MyD88–NF-κB-signaling pathway.

The gastrointestinal epithelial cells act as a physical barrier regulating the passive movement of ions, solutes, macromolecules, and microbes into the host (59). Intestinal permeability and barrier function are regulated by the expression of TJ proteins including occludin, claudin, and ZO-1 (59). Gut inflammation, such as IBD (60), coeliac disease (61), and ulcerative jejunitis (62) may lead to impaired gut-epithelial barrier function, thus causing the diffusion of pathogens, toxins, and allergens from the lumen into the circulatory system. In this study, we assayed for changes in TEER of the mouse jejunal epithelium, as an indicator of intestinal epithelial integrity and permeability (63). Both C-L and Enro were able to reverse EHEC-dependent changes in TEER, however, Enro had less of an effect on TEER values than C-L. Our western blot data revealed that EHEC infection decreased occludin and ZO-1 protein expression in the jejunum of mice. C-L effectively attenuated the EHEC-induced disruption of occludin and ZO-1 expression in the jejunum, whereas Enro only attenuated the disruption of ZO-1 expression. Collectively, C-L showed a stronger protective effect on the intestinal epithelial barrier function than Enro, which may be attributed to the better anti-inflammatory activity of C-L. In addition, immunofluorescence analysis revealed that C-L enhanced the abundance of TJ proteins and promoted ZO-1 localization to the intestinal epithelium. Consistent with these findings, TEM results also supported the protective effect of C-L against EHEC-induced impairment in jejunum tissues. Collectively, these data show that C-L protected barrier integrity by maintaining the expression of TJ proteins and reducing the severity of gut inflammation. Given the differential effects of C-L and Enro on the functions of the intestinal epithelial barrier and microbiota, we also investigated the defense of Enro- or C-L-treated mice against EHEC infection. Consistently, more bacteria were present in the spleen and liver of Enro-treated group than those of C-L-treated mice, suggesting that C-L-treated mice mounted a better defense against bacterial infection than antibiotic-treated mice.

## Conclusion

The present findings indicate that a novel hybrid peptide, C-L, designed in our laboratory effectively attenuated intestinal inflammation in EHEC O157:H7-infected mice. C-L treatment improved the microbiota composition and microbial community balance in mouse intestines. The hybrid peptide exhibited improved anti-inflammatory properties compared to Enro at the same concentration. Hybrid peptide treated infected mice demonstrated reduced clinical signs of inflammation, reduced weight loss, reduced expression of pro-inflammatory cytokines TNF-α, IL-6, and IFN-γ, reduced apoptosis, and reduced markers of jejunal epithelial barrier function. Our data suggest that the appropriate level of C-L treatment in animals may be 8–16 mg/kg. The anti-inflammatory potential of C-L could be exploited in technological and clinical applications, i.e., as antibacterial agents, healthcare formulas, or therapeutic anti-inflammatory drugs for animals or even humans.

## Data Availability Statement

All datasets generated for this study are included in the article/supplementary material.

## Ethics Statement

The animal study was reviewed and approved by Institutional Animal Care and Use Committee of China Agricultural University.

## Author Contributions

XW, LZ, RZ, MK, DS, and BA conceived and designed the experiments. XW, LZ, BA, JC, JW, MA, and MZ performed experiments and evaluated the data. XW prepared the manuscript and MK revised the manuscript. All authors assisted in the preparation of the manuscript and approved the final version.

## Conflict of Interest

The authors declare that the research was conducted in the absence of any commercial or financial relationships that could be construed as a potential conflict of interest.
